# Genome-based reclassification of Peribacillus castrilensis Rodríguez et al. 2023 as a later heterotypic synonym of Peribacillus frigoritolerans (Delaporte and Sasson 1967) Montecillo and Bae 2022, and an emended description of Peribacillus frigoritolerans

**DOI:** 10.1099/ijsem.0.006998

**Published:** 2025-12-19

**Authors:** Richard W. McLaughlin, Madisyn Myers, Mostafa S. Elshahed, Paul A. Lawson, Samuel L. Miller

**Affiliations:** 1 School of Liberal Arts and Sciences, Gateway Technical College, Kenosha, WI 53144, USA; 2 Department of Microbiology and Molecular Genetics, Oklahoma State University, Stillwater, OK 74074, USA; 3 School of Biological Sciences, University of Oklahoma, Norman, OK 73019, USA

**Keywords:** genome, heterotypic synonym, *Peribacillus*, *Peribacillus castrilensis*, *Peribacillus frigoritolerans*, taxonomy

## Abstract

A comparison of 16S rRNA gene sequences between *Peribacillus frigoritolerans* DSM 8801 ^T^ and *Peribacillus castrilensis* N3^T^ revealed 99.9% sequence similarity, suggesting their close relatedness and the possibility of these strains belonging to the same species. The genus *Peribacillus* includes 21 species with validly published names. The type species *P. frigoritolerans* DSM 8801^T^ (=ATCC 25097^T^=CCUG 43489^T^=CIP 67.20^T^=JCM 11681^T^) was isolated from arid soil in Morocco, while *P. castrilensis* N3^T^ (=CECT 30509^T^=LMG 32505^T^) was isolated from the faeces of a river otter in Castril (Granada, southern Spain). The present study used whole-genome data to clarify the taxonomic assignment of these two closely related *Peribacillus* species. *P. frigoritolerans* DSM 8801^T^ and *P. castrilensis* N3^T^ share similar chemotaxonomic and phenotypic characteristics. Their main cellular fatty acids were anteiso-C_15 : 0_ and iso-C_15 : 0_. Overall genomic relatedness indices analyses indicated that *P. frigoritolerans* DSM 8801^T^ and *P. castrilensis* N3^T^ shared average nucleotide identity and digital DNA–DNA hybridization values >95% and >70%, which are the currently accepted thresholds for species-level delineation. Additionally, these taxa formed a tight cluster within the genus *Peribacillus* in both the 16S rRNA gene phylogenetic tree and the core-genome phylogenomic tree. Based on the combined evidence and the earliest valid publication, priority is given to *P. frigoritolerans* (Delaporte and Sasson 1967) Montecillo and Bae 2022. *Peribacillus castrilensis* Rodríguez *et al*. 2023 is proposed to be a later heterotypic synonym of *P. frigoritolerans* (Delaporte and Sasson 1967) Montecillo and Bae 2022.

## Full text

The genus *Peribacillus* was proposed by Patel and Gupta [1] and currently includes 21 published species (https://lpsn.dsmz.de/genus/Peribacillus, October 2025). Members of this genus have been isolated from a variety of environments, such as faeces [2], soil [3,5], the Kennedy Space Center [6] and the internal stem tissue of a healthy cotton plant [7]. The type species of the genus was isolated from arid soil in Morocco [3]. This bacterium was named *Brevibacterium frigoritolerans* in the original publication but later transferred to the genus *Bacillus* [8] and subsequently reassigned to *Peribacillus* [9]. *Peribacillus frigoritolerans* DSM 8801^T^ is a Gram-stain-positive, strict aerobe, motile, spore-forming rod, capable of growth in 0.5–7.5% (w/v) NaCl and a pH range of 5.0–10.0 (optimum 7.0) [8]. Another *Peribacillus* strain, *Peribacillus castrilensis*, was isolated from the faecal material of a river otter in Spain [2]. *P. castrilensis* N3^T^ is a Gram-stain-positive, aerobic, motile, spore-forming rod, capable of growing in 0.5–7.5% (w/v) NaCl and a pH range of 6.0–8.0 (optimum 7.0) [2]. In both strains, the major cellular fatty acids were anteiso-C_15 : 0_ and iso-C_15 : 0_ [2].

**Fig. 1.**
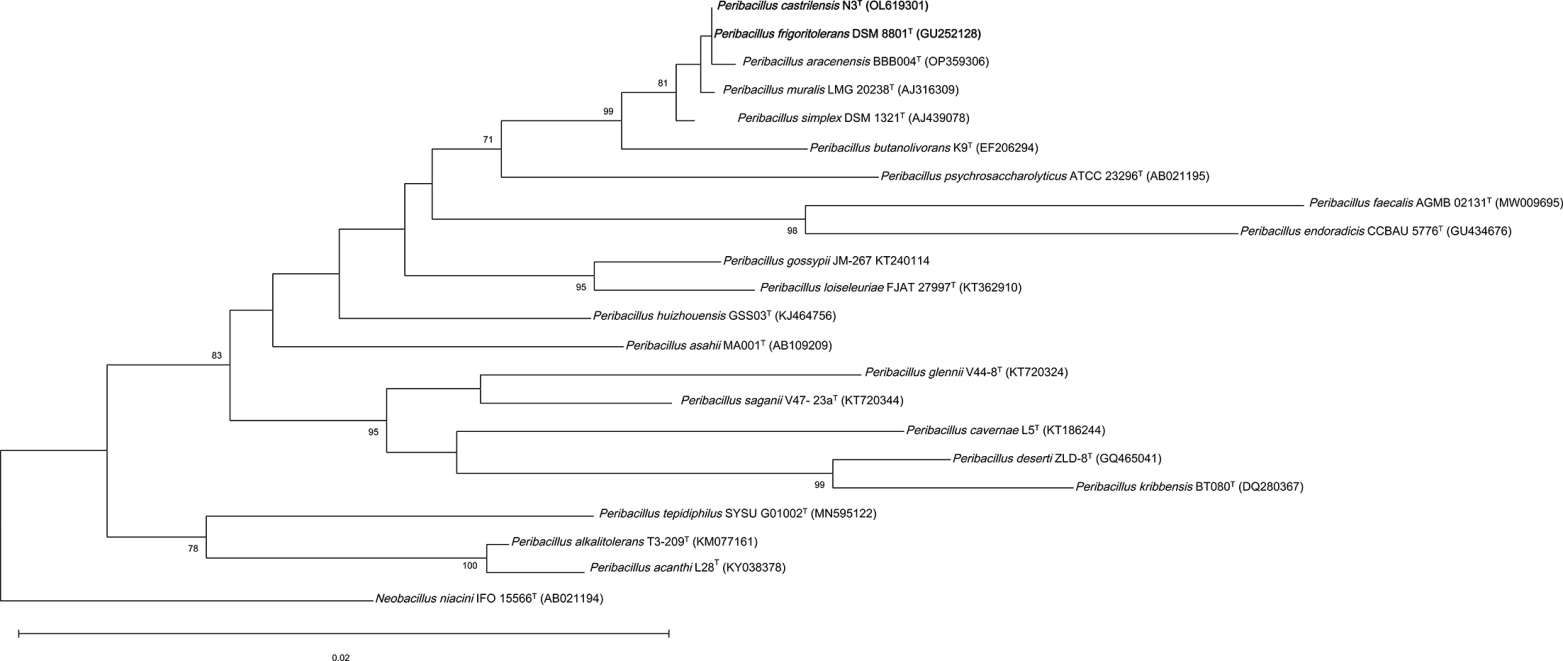
Phylogenetic tree based on 16S rRNA gene sequences showing the relationship among *P. frigoritolerans* DSM 8801^T^ and *P. castrilensis* N3^T^ compared to all other species with validly published names within the genus *Peribacillus*. The tree was constructed using the ML method. Bootstrap values expressed as percentages of 1,000 replications are given at branching points. Database accession numbers are given in parentheses. *Neobacillus niacini* NBRC 15566^T^ (AB021194) was used as an outgroup. Bar, 0.02 substitutions per nucleotide position.

Upon comparison, the 16S rRNA gene sequences of *P. frigoritolerans* DSM 8801^T^ (GU252128) and *P. castrilensis* N3^T^ (OL619301) were found to be 100.0% identical (Table 1), indicating an extremely close relationship, as this exceeds the 98.7% threshold value used in the assignment of strains to a species [10]. The EzBioCloud server [11] was then used to retrieve 16S rRNA gene sequences from all validly named members of the genus *Peribacillus*. ClustalW was then used to align the sequences [12]. A phylogenetic analysis was constructed in mega12 [13], using the maximum-likelihood (ML) algorithm [14] (Fig. 1). Genetic distances were then calculated using the Kimura two-parameter model [15]. Finally, bootstrap values were calculated using the standard 1,000 replications [16]. The phylogenetic tree based on the 16S rRNA genes also showed that *P. frigoritolerans* DSM 8801^T^ and *P. castrilensis* N3^T^ formed a distinct clade from other *Peribacillus* species (Fig. 1). The clade relationship was previously confirmed in the study by Rodríguez *et al*. [2].

**Table 1.** Pairwise comparison of 16S rRNA gene similarity and overall genomic relatedness indices between *P. frigoritolerans* DSM 8801^T^, *P. castrilensis* N3^T^ and their close relatives

16S rRNA gene similarity (%)			
**Taxa**	16S rRNA gene accession	***P. castrilensis***	***P. frigoritolerans***
***P. castrilensis***	**OL619301**	**100.0**	**100.0**
***P. frigoritolerans***	**GU252128**	**100.0**	**100.0**
*P. aracenensis*	OP359306	99.9	99.9
*P. butanolivorans*	EF206294	99.1	99.1
*P. muralis*	AJ316309	99.9	99.9
*P. psychrosaccharolyticus*	AB021195	98.2	98.2
*P. simplex*	AJ439078	99.9	99.9
**dDDH (%)**			
**Taxa**	**WGS accession**	***P. castrilensis***	***P. frigoritolerans***
***P. castrilensis***	**GCA_021012855.1**	**100.0**	**74.6**
***P. frigoritolerans***	**GCA_024169475.1**	**75.1**	**100**
*P. aracenensis*	GCA_025175645.1	48.8	48.4
*P. butanolivorans*	GCF _001273755.1	29.5	29.4
*P. muralis*	GCF _001439925.1	29.1	29.2
*P. psychrosaccharolyticus*	GCA_000305495.2	19.7	21.2
*P. simplex*	GCA_002243645.1	53.2	52.1
**ANI (%**)			
**Taxa**	**WGS accession**	***P. castrilensis***	***P. frigoritolerans***
***P. castrilensis***	**GCA_021012855.1**	**100.0**	**97.0**
***P. frigoritolerans***	**GCA_024169475.1**	**97.0**	**100.0**
*P. aracenensis*	GCA_025175645.1	92.7	92.5
*P. butanolivorans*	GCF _001273755.1	84.8	84.6
*P. muralis*	GCF _001439925.1	84.8	84.6
*P. psychrosaccharolyticus*	GCA_000305495.2	77.3	77.2
*P. simplex*	GCA_002243645.1	93.7	93.4
**AAI (%)**			
**Taxa**	**WGS accession**	***P. castrilensis***	***P. frigoritolerans***
***P. castrilensis***	**GCA_021012855.1**	**100.0**	**97.2**
***P. frigoritolerans***	**GCA_024169475.1**	**97.2**	**100**
*P. aracenensis*	GCA_025175645.1	93.5	93.5
*P. butanolivorans*	GCF _001273755.1	87.1	87.2
*P. muralis*	GCF _001439925.1	86.4	86.7
*P. psychrosaccharolyticus*	GCA_000305495.2	67.8	67.7
*P. simplex*	GCA_002243645.1	94.7	94.4

Strains: *P. castrilensis* N3T; *P. frigoritolerans* DSM 8801T; *P. aracenensis* BBB004T; *P. butanolivorans* K9T; *P. muralis* LMG 20238T; *P. psychrosaccharolyticus* ATCC 23296T; *P. simplex* DSM 1321T.

**Fig. 2.**
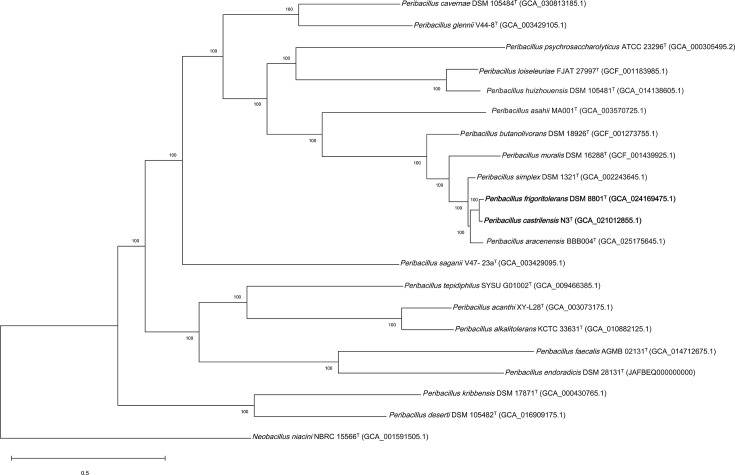
Core-genome phylogenomic tree using a concatenated alignment of 672 single-copy genes showing the relationship among *P. frigoritolerans* DSM 8801^T^ and *P. castrilensis* N3^T^ among other species of the genus *Peribacillus*. The tree was constructed using Codon Tree. Database accession numbers are given in parentheses. *N. niacini* NBRC 15566^T^ (GCA_001591505.1) was used as an outgroup. Bar indicates the mean number of substitutions per site, 0.5.

The genomes of *P. frigoritolerans* DSM 8801^T^ and *P. castrilensis* N3^T^ have been sequenced (GenBank accession numbers GCA_024169475.1 and GCA_021012855.1, respectively). To further explore the phylogenetic relationship between *P. frigoritolerans* DSM 8801^T^ and *P. castrilensis* N3^T^, average nucleotide identity (ANI) and average amino acid identity (AAI) between the two micro-organisms and their close relatives was calculated [17] (Table 1). An ANI value of 97.0% was obtained between both organisms, exceeding the proposed species cut-off boundary for ANI (95%–96% [18]). An AAI value of 97.2 was observed between both organisms (Table 1). These high levels of relatedness were further confirmed using Type (Strain) Genome Server [19], which showed that both genomes were derived from the same species with a digital DNA–DNA hybridization (dDDH) value of 75.1% (Table 1). Finally, to further confirm the close taxonomic relationship between both strains, a core gene phylogenomic tree was constructed using the Codon Tree Method [20] (Fig. 2). The phylogenomic tree showed a close relationship between *P. frigoritolerans* DSM 8801^T^ and *P. castrilensis* N3^T^ (Fig. 2).

In the *P. castrilensis* description manuscript [2], both strains were compared. Biochemical and physiological characterization efforts identified a high level of similarity with only a few differences (summarized in Table 2). The paper also showed a high level of sequence similarity to generate a phylogenetic tree displaying such a close relationship. However, a decision to propose a new species *P. castrilensis* to accommodate strain N3^T^ was taken based on incorrectly calculated ANI and dDDH values. Here, we argue that, considering the few biochemical and physiological discrepancies reported, the high sequence similarity metrics and the high phylogenetic congruence observed using the 16S rRNA gene (Fig. 1) and core-genome phylogenies (Fig. 2), *P. frigoritolerans* DSM 8801^T^ and *P. castrilensis* N3^T^ belong to the same species.

**Table 2.** Differential physiological characteristics for *P. frigoritolerans* DSM 8801^T^ and *P. castrilensis* N3^T^

Characteristic	*P. castrilensis*	*P. frigoritolerans*
**Indole**	+	−
**Gelatin hydrolysis**	+	−
**Acid production from**		
**Arabinose**	−	+
**Fructose**	−	+
**Glucose**	−	+
**Lactose**	+	−
**Raffinose**	−	+
**Sucrose**	−	+

Data were taken from [2]. +, positive; −, negative. The following characteristics were shared between both isolates: strict aerobe (+), endospores (+), motility (+), maximum NaCl for growth (%, w/v) 7.5, oxidase (−) and urease (−); acid production from citrate (−), galactose (−), glycerol (−), inulin (−), mannitol (−) and trehalose (−).

Per the priority of prokaryotic names governed by the International Code of Nomenclature of Prokaryotes [21], the earliest validly published name is used for the union when species are united. Therefore, *P. castrilensis* Rodríguez *et al.* 2023 is proposed to be a later heterotypic synonym of *P. frigoritolerans* (Delaporte and Sasson 1967) Montecillo and Bae 2022.

## Emended description of *Peribacillus frigoritolerans*

The description is as before [2] with the following modifications.

The type strain is DSM 8801^T^ (=ATCC 25097^T^=CCUG 43489^T^=CIP 67.20^T^=JCM 11681^T^), and strain N3 (=CECT 30509=LMG 32505) is an additional strain. The additional strain of *P. frigoritolerans* shows that this species has an optimum growth in 1.0% NaCl with a range of 0.5–7.5% and a pH range of 6.0–8.0 (optimum 7.0). The bacterium does not grow at 4 °C. Starch, casein, gelatin hydrolysis and nitrate reduction are variable. Both arginine dihydrolase and tryptophan deaminase production are variable. Acid production from cellobiose, fructose, glucose, inositol, l-arabinose, mannose, raffinose and sucrose is also variable.

The emended DNA G+C content (mol%) is 40.3–40.6. The genome sequence was deposited in GenBank under the accession number GCA_021012855.1. The genome size is 5.7 Mb.

## Abbreviations

AAI: average amino acid identity
ANI: average nucleotide identity
dDDH: digital DNA–DNA hybridization
ML: maximum-likelihood
